# Disseminated Invasive Mucormycosis Infection Following Autologous Stem Cell Transplantation for Diffuse Large B-Cell Lymphoma

**DOI:** 10.1007/s44228-023-00031-z

**Published:** 2023-02-08

**Authors:** Edward R. Scheffer Cliff, Gemma Reynolds, Andrew Grigg

**Affiliations:** 1 Department of Clinical Haematology, Austin Health, Heidelberg, VIC Australia; 2 Department of Infectious Diseases, Austin Health, Heidelberg, VIC Australia; 3 Department of Infectious Diseases, Peter MacCallum Cancer Centre, Parkville, VIC Australia; 4 Department of Medicine, University of Melbourne, Parkville, VIC Australia; 5 Olivia Newton-John Cancer Research Institute, Austin Health, Heidelberg, VIC Australia

**Keywords:** Mucormycosis, Mucor, Invasive fungal infection, Lymphoma, Autologous stem cell transplant

## Abstract

Invasive fungal infections (IFI) are challenging to predict, diagnose and treat, and are associated with a particularly high mortality among patients with hematological malignancies. They are relatively uncommon in patients with lymphoma, compared with those with acute leukemia or undergoing allogeneic transplantation. We present a patient, autografted for recurrent lymphoma, with fever and refractory diarrhea persisting post engraftment, eventually attributable to disseminated mucor infection. This case illustrates the challenge of timely diagnosis and initiation of treatment for IFI in lymphoma patients, who do not routinely receive antifungal prophylaxis, and the importance of aggressive investigation and symptom-directed tissue sampling for evidence of IFI in febrile immunocompromised hosts not responding to broad-spectrum antibiotics.

## Introduction

Invasive mucormycosis (IM) is a rapidly-progressive, angioinvasive fungal infection (IFI) with very high mortality. In hematology patients, IM has been classically associated with acute myeloid leukemia and allogeneic stem cell transplantation and its complications, particularly Graft-versus-Host-Disease (GvHD), prolonged neutropenia, high-dose corticosteroids, iron overload, fludarabine exposure, and Aspergillus-directed prophylaxis such as voriconazole [1–5]. Broader risk is also conferred by diabetes and older age [2]. IM has recently been associated with COVID-19 infection [6]. Among hematology patients, IM incidence ranges between 0.09 and 8%, depending on host risk factors, underlying hematological disease and prophylaxis strategy [4, 7, 8]. The lungs (30–50%) and sinuses (20–40%) are the most common sites of disease involvement, with 10–15% of patients presenting with rhinocerebral or disseminated disease [2]. IM seldom involves the gastrointestinal system, representing only 4% of reported cases [2].

IM is, however, extremely rare in patients with lymphoma, including autologous stem cell transplantation (autoSCT) recipients, with a post-autoSCT incidence < 0.1% [4, 7]. Here we report a case of fatal, disseminated gut mucormycosis in a 62-year-old male following autoSCT for diffuse large B-cell lymphoma (DLBCL).

## Case

A 62-year-old Italian-born Australian man was diagnosed in 2016 with asymptomatic small lymphocytic lymphoma (SLL) with extensive marrow involvement without peripheral blood lymphocytosis. Cytogenetics demonstrated trisomy 12. After 4 years of observation, he presented with a subcutaneous lesion, biopsy of which demonstrated Richter’s transformation to a composite follicular grade 3A/DLBCL (Ki67 70% in the diffuse areas). Positron Emission Tomography/Computed Tomography (PET/CT) scan showed multiple nodes of low avidity consistent with SLL. His past medical history was notable for a chronic left mastoid cavity with recurrent sinusitis in childhood, obesity (Body Mass Index > 35), non-alcoholic non-cirrhotic fatty liver disease, a significant smoking history and banded hemorrhoids.

Prior to commencing R-CHOP (rituximab, cyclophosphamide, vincristine, doxorubicin and prednisolone), he experienced self-resolving antibiotic-associated diarrhea, negative for *Clostridioides difficile* on polymerase chain reaction (PCR). At diagnosis, immunoglobulin levels were normal (IgG 9.96 g/L [normal range 7–16 g/L], IgA 1.62 g/L [0.7–4], IgM 0.47 g/L [0.4–2.3]). Prophylaxis with entecavir was given due to the risk of hepatitis B reactivation (HbsAg negative, anti-HbcAb positive).

After 3 cycles of R-CHOP, a repeat PET/CT showed an area of high avidity in the neck, biopsy of which showed DLBCL of germinal center type (BCL-2 positive, MYC negative on immunohistochemistry; Fluorescence In Situ Hybridization (FISH) not performed). Complete remission was demonstrated on PET/CT following salvage therapy with rituximab, ifosfamide, carboplatin and etoposide (R-ICE) and he subsequently underwent an autoSCT with standard-dose BEAM (carmustine, etoposide, cytarabine, melphalan) conditioning. He received neither antibiotic nor antifungal prophylaxis.

Day 1 post-transplant, non-bloody diarrhea developed, with 7–10 bowel movements per day. Absolute neutropenia (ANC < 0.5 × 10^9^/L) developed on Day 3, with culture-negative febrile neutropenia on Day 4. Intravenous (IV) piperacillin-tazobactam 4.5 g six-hourly was commenced. Granulocyte colony-stimulating factor 480 μg daily was given from days 5–9. Fecal samples on days 0, 5, 12 and 19 were negative on culture and so was PCR for bacterial, viral and parasitic pathogens. CT on D6 demonstrated non-specific, mild, inflammatory stranding around the proximal small bowel. Neutrophil recovery to 1.1 × 10^9^/L occurred on Day 9 and remained > 3 × 10^9^/L thereafter. Persistent fever > 38.0 °C (without hemodynamic instability) were attributed to engraftment syndrome and treated with 100 mg prednisolone for 4 days.

**Fig. 1:**
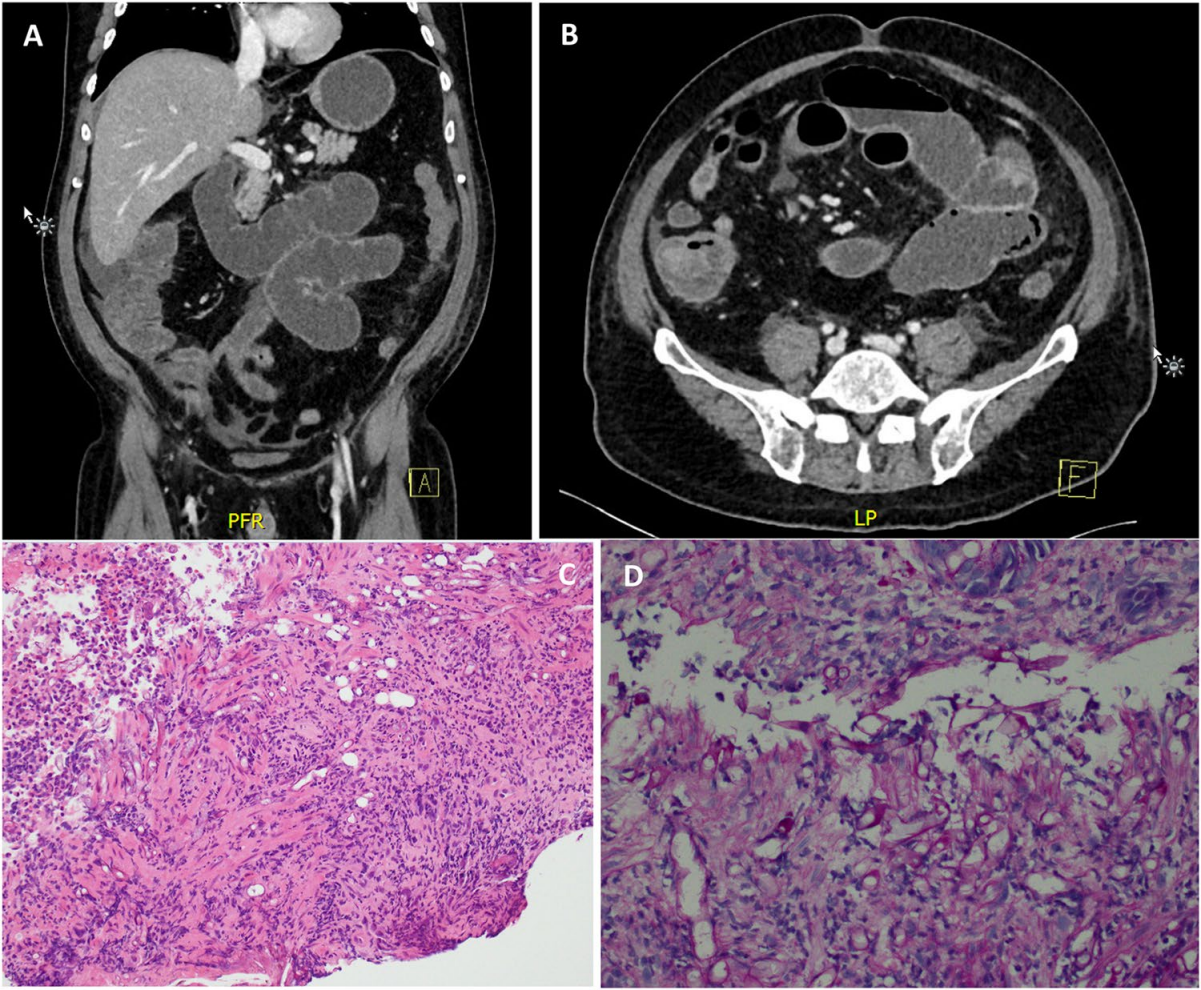
**A**, **B** Computed tomography of the abdomen and pelvis, with contrast, demonstrates a multifocal enterocolitis with interval development of severe small bowel dilatation with a progressive transition zone in the inflamed distal ileum. **C**, **D** Histopathology demonstrates areas of ulceration with necrotic debris and inflammatory exudate, showing multiple right-angle branching and broad ribbon shaped fungal elements within the exudate as well as inflamed mucosa, involving lamina propria, crypts and underlying muscularis mucosae

Post engraftment, profuse watery diarrhea continued, with associated crampy abdominal pain and fever. Day 14 sigmoidoscopy demonstrated macro- and microscopically normal colonic mucosa, although this biopsy was not taken from the radiologically-involved bowel, which was right-sided. Repeat CT on Day 19 demonstrated interval development of multifocal enterocolitis with severe small bowel dilation (Fig. 1A, B). Ileus was managed with nasogastric and rectal decompression and total parenteral nutrition.

Full colonoscopy on Day 20 demonstrated mucosal ulceration, predominantly in the ascending and proximal transverse colon. Histopathology demonstrated ulceration with necrotic debris and inflammatory exudate, and embedded right-angle branching and broad ribbon-shaped fungal elements (Fig. 1C, D). The branching pattern and a positive PAS-D staining pattern were suggestive of mold. PCR of colon tissue detected *Rhizopus microsporus* DNA, but this result was only available on Day 40, a week after the patient’s death. Day-30 PET/CT demonstrated diffuse small bowel and pulmonary uptake, consistent with disseminated fungal infection, despite a lack of pulmonary symptoms.

Combination intravenous liposomal amphotericin B (5 mg/kg daily) and IV posaconazole 300 mg daily (with BD loading) commenced on Day 20. IV immunoglobulin was given on Day 26 for panhypogammaglobulinemia (IgG 2.24 g/L, IgM 0.21 g/L, IgA < 0.2 g/L). Concomitant low-level cytomegalovirus (CMV) viraemia was identified on Day 19 (viral load < 200 copies/mL). IV ganciclovir was commenced on Day 21 (PCR result showed 1661 copies/mL) amid clinical concern for CMV colitis pending histopathology. Subsequent CMV immunohistochemistry was negative on Day 20 samples, and no viral inclusions were seen. Despite combination antifungals, the patient deteriorated and died on Day 33. No autopsy was performed.

## Discussion

This case highlights the difficulty in predicting, preventing, and treating IM in hematology patients outside the usual contexts of GvHD post-allograft or prolonged neutropenia during acute leukemia induction treatment. Among autoSCT patients, risk factors for IFI in general include the number of treatment lines, age > 60 years, and duration of neutropenia [8] but, due to its rarity, specific risk factors for IM have not been identified. Across systematic reviews examining IM in immunocompromised hosts, up to 25% of patients had no classical risk factors [9, 10], although the most frequently reported across a broader cohort of patients include corticosteroid therapy, followed by diabetes, blood cancers, and organ transplantation. In our review of proven *Mucorales* cases amongst lymphoma patients across PubMed and EMBASE, only 3 of 21 patients had undergone autoSCT, and typical IFI risk factors including prolonged neutropenia and prolonged steroid exposure were uncommon (Table 1).

**Table 1:** EORTC-proven cases of Mucorales infections in adult patients with lymphoma where patient-level data is provided

Case	Age Gender	Hematologic diagnosis	Prior therapy	HSCT	Anti-mold prophylaxis	Timing	Fungus	Primary site	Disseminated	Management	Outcome
1 [17]	46F	T-lymphoblastic lymphoma	2 cycles Hyper-CVAD	No	No	During C2	*Lichtheimia corymbifera*	Pulmonary	Yes (renal)	L-AMB + Posaconazole	Alive, 18 months
2 [18]	32M	Stage IV B-cell NHL	AVBD	No	No	NR	*Fusariam moniliforme*	Cutaneous	Yes (blood, lungs)	NR	Died of IFI, day 15
3 [19]	69F	Relapsed follicular lymphoma	6 × R-CHOP, 2 × R-DeVIC	No	No (Fluconazole)	C3 R-DeVIC	*Exophiala dermatiditis*	Pulmonary	Yes (blood)	L-AMB (1/7) + Voriconazole (NR)	Alive, 12 months
4 [20]	62F	Stage IV DLBCL	6 × R-CHOP	Autologous	No	12 months post HSCT	*Scedosporium angiosporum*	Cutaneous	No	Voriconazole	Alive, 2 months
5 [21]	78M	Lymphoplasmacytic lymphoma	Chlorambucil (24/12), 2 × CDA	No	No	During C2 CDA	Mucorales (histological)	Unknown	Yes (multi-organ)	Nil anti-fungal therapy	Died of IFI, day 122
6 [22]	56M	Post-transplant DLBCL (pancreas /kidney transplant)	R-ICE + Ibrutinib	No	No	1/12 post starting Ibrutinib	*Rhizomucor*	Hepato-splenic	Yes	L-AMB, liver resection, splenectomy, cholecystectomy	Alive, 12 months (isavuconazole ongoing)
7 [23]	35F	High-grade B-cell Lymphoma	R-CHOP, CODOX-M-IVAC	No	No	End of CODOX	Mucorales (histological)	Rhino-cerebral	No	L-AMB + Posaconazole	Died of lymphoma, 3 months
8 [24]	36F	T-cell lymphoma	7 × Etop/Carbo/ Cytarabine, RTx	No	No	After RTx	*Syncephalastrum racemosum*	Pulmonary	No	L-AMB + itraconazole, caspofungin, voriconazole	Died of lymphoma, 10 months
9 [25]	42F	Follicular lymphoma + T-cell lymphoma	Fluda, CHOP, R, chlor, ESHAP	No	No	C1 ESHAP	Mucorales (histological)	CNS	Yes (multi-organ)	L-AMB	Died of IFI, day 23
10 [25]	63M	Stage IV DLBCL	R-EPOCH	No	No	C1D15	*Rhizopus microsporus*	Pulmonary	Yes (multi-organ)	L-AMB	Died of IFI, day 54
11 [26]	66M	Mantle cell lymphoma	6 cycles R-CHOP	No	No	Unknown	*Rhizopus microsporus*	Cutaneous	No	L-AMB	NR
12 [27]	55M	Stage IV DLBCL	3 cycles R-CHOP	No	No	After Cycle 3	*Paecilomyces variotti*	Unknown	Yes	L-AMB	Alive, 6 weeks
13 [28]	63M	Mantle cell lymphoma	1 cycle R-CHOP	No	No	Cycle 1	Mucorales (histological)	Unknown	Yes (autopsy)	Nil	Died of IFI, day 17
14 [29]	38F	T-lymphoblastic lymphoma	GMALL protocol	No	No	Cycle 1	Mucorales (histological)	Brain	No	Nil	Died of IFI, day 43
15 [30]	49M	Relapsed NHL	‘chemotherapy’	No	No	NR	Mucorales (histological)	Unknown	Yes: lung, liver, kidney	Nil	Died of IFI
16 [31]	20M	NHL	Cytarabine	No	No	NR	Mucorales (histological)	Unknown	Yes: Lung, heart, liver, brain, kidney	L-AMB	Died of IFI
17 [32]	23M	Hepatosplenic T-cell lymphoma with HLH	ESHAP, CHOP	No	No	Post C1 CHOP	Mucorales (histological)	Lung	Yes (autopsy)	L-AMB	Died day 10, progressive lymphoma, also IFI on autopsy
18 [33]	57F	Refractory DLBCL	Cyclo-dex, alemtuzumab	No	Itraconazole	D10 post alemtuzumab	*Mucor circinelloides* + *IA*	Periorbital	No	L-AMB + voriconazole	Died of lymphoma, 1 month
19 [34]	42M	Anaplastic large cell lymphoma	CHOP + RTx. + BEAM ASCT	Autologous	No	D100 HSCT	Mucorales (histological)	Spleen	Liver, spleen	Amphotericin, itraconazole	Alive, > 11 months
20 [35]	31F	DLBCL	NR	NR	No	NR	*Rhizopus microsporus*	Lung	No	Voriconazole, micafungin (empirical)	Died of IFI, 1 month
21 [36]	74M	Mantle cell lymphoma	NR	Autologous	NR	D48 post HSCT	Mucorales (histological)	GI	No	L-AMB	Died of lymphoma, 10 months

Studies in languages other than English and in patients with AIDS excluded

*C* cycle, *D* day, *M* male, *F* female, *NHL* non-Hodgkin lymphoma, *DLBCL* diffuse large B-cell lymphoma, *L-AMB* liposomal amphotericin B, *IFI* invasive fungal infection, *NR* not reported, *RTx* radiotherapy, *HLH* Hemophagocytic Lymphohistiocytosis, *IA* invasive aspergillosis, *GI* gastro-intestinal, *EORTC* European Organisation for Research and Treatment of Cancer

Our case had no clearly identifiable risk factors. He had received only two prior chemotherapy regimens prior to autograft and had never experienced prolonged neutropenia. In subsequent discussions with his family, there was no obvious prior environmental exposure to mucor, which has reported associations with contaminated food (such as fermented dairy products, fermented porridges and dried breads), naturopathic medicines and medical supplies [11]. There were no concomitant episodes of mucor at our institution. Other than sinusitis, our patient had no features to suggest congenital immunodeficiency, although he did have underlying SLL, which is inherently immunosuppressive [12]. Although enteral nutrition is associated with fungal infection, symptoms developed prior to its initiation. The high dose corticosteroid therapy as treatment for apparent engraftment syndrome was given after the development of symptoms, thus unlikely to be a primary contributing factor, although its immunosuppressive effect may have increased the risk of dissemination.

Of note, the patient had CMV reactivation, which has been associated with IFI [13], including after autoSCT [8], although the nature of the association remains unclear. While the immunosuppressive effect of CMV—impaired macrophage migration and antigen presentation – may play a role, CMV and IFI may simply both occur in the most severely immunosuppressed patients. The chronology of this case with low level CMV reactivation after the likely onset of IM would argue against it being a contributing factor. In summary, it is likely that an unidentified environmental exposure led to invasive infection in the context of multi-modal immunosuppression, on a background of SLL.

Could the diagnosis have been made earlier? The combination of persistent diarrhea and fever post-engraftment, resistant to steroids, should raise suspicion for opportunistic gastrointestinal infection. Aggressive and prompt investigation with endoscopic biopsies and multidisciplinary correlation of radiological, microbiological, and histological findings is critical to the diagnosis. The extent of endoscopy evaluation should be guided by imaging close to the procedure. Of note, in this case, a limited sigmoidoscopic biopsy was performed 8 days after the most recent CT, by which time it is likely that progression to colonic involvement had occurred. This may have been a missed opportunity for earlier intervention; the definitive diagnostic procedure and commencement of therapy occurred 6 days later, by which time the disease was irreversibly severe.

Histological evidence of fungal hyphae is particularly crucial for diagnosis, as molecular confirmation typically takes weeks. In a recent systematic review of gastrointestinal mucor, the diagnosis was made by histology in 49 of 70 cases (70%), combined PCR and culture in 8 (11%) and PCR and histology in 5 (7%), culture in 3 (4%), and combined histology, PCR and culture in 5 (7%) [9]. To date, there are no proven serum-based rapid diagnostic tests for IM. Although quantitative PCR detection of *Mucorales* DNA in serum is a more rapid method of IM detection [14], it is challenging to widely validate, given the rarity of IM.

Surgical resection remains the mainstay of IM treatment, but is futile in patients with disseminated disease; extensive gastrointestinal mucor is typically a harbinger of dissemination [15]. The decision not to pursue resection in our patient was based on clinical suspicion of dissemination. There are limited data on the efficacy of medical treatment alone in isolated gastrointestinal IM: in the aforementioned review, 13 of 21 patients who did not receive surgery survived [9]. It is likely that the localization of the initial infection (for example a discrete esophageal mass rather than the extensive bowel involvement in this case) and the depth and reversibility of immunosuppression are intricately linked to the ability to survive IFI without resection.

Medical treatment requires mold-active anti-fungal therapy, of which liposomal amphotericin, posaconazole and isavuconazole are the licensed therapeutic options with in vitro activity [5]. Therapy should be started as soon as there is histological suspicion of mucor without waiting for molecular confirmation. Where relevant, other measures such as correction of hypogammaglobulinemia and cessation or minimization of immunosuppression should be implemented but, as this case suggests, may not have a substantive impact on outcome. Although combination antifungal therapy offers the possibility of increased efficacy in IM, a multi-center retrospective study demonstrated only a nonsignificant trend towards reduction in mortality when posaconazole or isavuconazole was added to liposomal amphotericin [3]. Novel antifungals have not yet made significant progress against *Mucorales* [5].

Current guidelines do not recommend the routine use of anti-mold prophylaxis in autoSCT recipients [1, 16], although one study suggested that, due to cumulative risk, prophylaxis may be justified in lymphoma patients aged over 60 years, who are undergoing autoSCT after ≥ 3 lines of chemotherapy [8]. Our patient would not have qualified using these criteria. Pre-transplant measurement of immune dysfunction may be useful, but without practical and reliably predictive ways to do so, and given the rarity of IFI in this context, this approach remains hypothetical.

In the absence of reliable predictive models, prompt diagnosis, which may improve outcome, relies initially on clinical suspicion. While diarrhea due to direct mucosal toxicity of high dose chemotherapy and neutropenic typhlitis is not uncommon amongst autoSCT patients, persistent and worsening culture-negative diarrhea post-engraftment with fever should raise suspicion that something unusual may be amiss.

## Data Availability

Data and materials are available for review upon reasonable request.
